# Investigation of the apoptotic way induced by digallic acid in human lymphoblastoid TK6 cells

**DOI:** 10.1186/1475-2867-12-26

**Published:** 2012-06-11

**Authors:** Wissem Bhouri, Jihed Boubaker, Ines Skandrani, Kamel Ghedira, Leila Chekir Ghedira

**Affiliations:** 1 Laboratoire de biologie Cellulaire et Moléculaire, Faculté de Medecine Dentaire Monastir, Rue Avicenne, Monastir, 5000 Tunisia; 2 Unité de pharmacognosie 99/UR/07, Faculté de pharmacie Monastir, Rue Avicenne, Monastir, 5000 Tunisia; 3 Faculty of Dental Medicine, Rue Avicenne, Monastir, 5019, Tunisia

**Keywords:** Caspase activity, DNA fragmentation, Digallic acid, PARP

## Abstract

**Background:**

The digallic acid (DGA) purified from *Pistacia lentiscus*. L fruits was investigated for its antiproliferative and apoptotic activities on human lymphoblastoid TK6 cells.

**Methods:**

We attempt to characterize the apoptotic pathway activated by DGA. Apoptosis was detected by DNA fragmentation, PARP cleavage and by evaluating caspase activities.

**Results:**

The inhibition of lymphoblastoid cell proliferation was noted from 8.5 μg/ml of DGA. The induction of apoptosis was confirmed by DNA fragmentation and PARP cleavage. We have demonstrated that DGA induces apoptosis by activating the caspase-8 extrinsic pathway. Caspase-3 was also activated in a dose dependent manner.

**Conclusion:**

In summary, DGA exhibited an apoptosis inductor effect in TK6 cells revealing thus its potential as a cancer-preventive agent.

## Background

Polyphenols were described to be beneficial against human diseases such as cancer and metastasis [1,2]. It has been shown, in both *in vitro* test and small animal model study, that polyphenols induce responses consistent with the protective effects of diets rich in fruits and vegetables against degenerative conditions such as cardiovascular disease and carcinogenesis [3,4]. Furthermore, they can be obtained without prescriptions. For example, fresh green tea contains large amounts of catechin polyphenol, while flavonoids resveratrol and quercetin are important in grapes, red wine, and other food products [5]. Epidemiologic studies suggest that diet can affect the risk for cancer and, particularly a diet rich in vegetables reduces this risk [6,7]. Apoptosis, or programmed cell death, is an important physiologic process in the normal development [8], and induction of apoptosis is a highly desirable mode as a therapeutic strategy for cancer control [9,10]. The major challenge in treating cancer is that many tumor cells carry mutations in key apoptotic genes such as p53, BCL family protein, or those affecting caspase signaling [11]. The BCL-2 family determines the life-or-death of a cell by controlling the release of mitochondrial apoptogenic factors associated with death proteases called caspases which are considered as a central player for the apoptotic process and cascade of proteolytic cleavage events [12]. In addition to its importance in the treatment of cancer, caspase-dependent processes have also been demonstrated to play an essential role in mediating cell death [13].

Caspase-dependent processes are associated with two pathways of anti-cancer drug-induced apoptosis, death receptor-dependent and mitochondria-dependent pathways [14]. The death receptor apoptosis pathway or the extrinsic pathway had been thought to involve only the caspase cascade in which caspase-8 activated the downstream effector caspases, such as caspase-3 and as such mitochondria pathway was not considered to be involved.

However, when it was found that caspase-8 could cleave Bid and the truncated Bid could then translocate to the mitochondria, it became clear that mitochondria-initiated apoptosis pathway (the intrinsic pathway) could also play a role in the death receptor-initiated apoptosis [15-17].

Death receptor activation pathway is mediated with a death-inducing signaling complex, which is made of a Fas-associated death domain and a procaspase-8, activating Caspase-8 [18]. Caspase-8 directly activates Caspase-3, leading to apoptosis.

Although many studies on the anti-cancer mechanisms of polyphenols have been reported, anti-cancer mechanism of digallic acid mediated caspases pathways have not been fully studied.

In the present study, the apoptotic effects of digallic acid (DGA), from *Pistacia lentiscus*. L fruits, on human lymphoblastoid cells TK6 were examined to determine the apoptotic pathway.

## Material and methods

### Chemicals

Dimethylsulfoxide (DMSO) was purchased from Sigma (St. Louis, MO, USA), RPMI-1640 Glutamax, foetal bovine serum and gentamicin were bought from GIBCO BRL Life technologies (Grand Island, NY, USA). The proteinase K, the ethylene diamine tetraacetic (EDTA), the sodium dodecyl sulfate (SDS), RNase A and MTT (3-(4,5-dimethylthiazol-2-yl)-2,5-diphenyltetrazolium bromide) were obtained from Sigma Aldrich Co (St. Louis, MO, USA). Acrylamide and bisacrylamide were purchased from (Madison,WI,USA), Ethidium bromide (EtBr) and Bromophenol blue were purchased from Merck (Darmstadt, FR, Germany). Agarose and Ployvinylidene difluoride (PVDF) membranes were obtained from Invitrogen, life technologies (UK). The monoclonal antibody i.e. anti poly ADP-ribose polymerase (anti-PARP) and the goat anti mouse alkaline phosphtase conjugated antibody were purchased from (St,Louis, Missouri, USA). The 5-Bromo-4Chloro-3 Indolyl Phosphate (BCIP)/Nitro Bleu Tetrazolium (NBT) and Tween 20 were purchased from promega (Madison, WI, USA). Caspase-3 and caspase-8 colorimetric assay kits were obtained from Sigma RBI (St Louis,MO,USA).

### Extraction method

**Figure 1:**
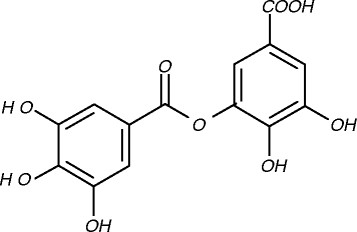
Chemical structure of Digallic acid.

The powdered fruits of *Pistacia lentiscus* were extracted with boiling water for 15– 20 min. After filtration, the extract was lyophilized leading to an aqueous one. The residue was suspended in water and successively portioned between water and chloroform, ethyl acetate and 1-butanol. Each liquid–liquid extraction was carried out three times (water: organic solvent = 1:1 v/v). The solvents of the obtained sub-extracts were evaporated under vacuum to dryness. The ethyl acetate soluble fraction (2 g) was fractionated over silica gel column eluted with CH_2_Cl_2_–MeOH gradually increasing the MeOH content and three fractions were collected. Fraction 1 was rechromatographed over Sephadex LH20 eluted with 100% MeOH and nine sub-fractions were obtained. Sub-fraction 7 was further purified over Sephadex LH20 column eluted with MeOH–H_2_O (9:1) and nine sub-fractions were obtained. Sub-fraction 6 was purified by passage through (C18) disposable extraction column eluted with methanol and water content to afford 24.5 mg of digallic acid (DGA). Figure 1[19].

### Nuclear magnetic resonance (NMR)

NMR spectroscopy experiments on the compounds were performed on a Bruker_Avance 400 at 400 MHz (for ^1^ H NMR) and 100 MHz (for ^13^ C NMR) with CD3OD as solvent. FAB–MS (negative- ion mode, glycerol matrix) was recorded on an R210C (VG Instruments, Altrincham, UK) spectrometer equipped with an IPC (P2A) MSCAN WALLIS computer system. COSY, HMQC, and HMBC spectra were obtained using the usual pulse sequences.

### Cell lines, cell culture and chemicals

Human lymphoblastoid cell line TK6 (Kindely provided by Pr. Pierre Biscoff centre Paul Strauss Strasbourg. France) expresses wild-type p53, and is thus p53 proficient. Cells were cultivated in RPMI-1640 glutamax supplemented with 10% (v/v) foetal bovine serum, 1 mM sodium pyruvate, 1 mM non-essential amino acids, 50 μg/ml gentamicin at 37°C in humidified atmosphere with 5% CO_2_. The experiments were performed after approximately two passages to limit chromosome instability due to culture maintenance.

### Cell proliferation assay

The evaluation of antiproliferative activity is based on the reduction of MTT (3-(4,5-dimethylthiazol-2-yl)-2,5-diphenyltetrazolium bromide) by the mitochondrial dehydrogenase of viable cells to give a blue formazan product which can be measured spectrophotometrically. The MTT colorimetric assay was performed in 96-well plates [20,21]. TK6 cells were seeded in 96 wells plate at a concentration of 5.10^4^ cells/well and incubated during 24 h at 37°C. After treatment with various concentrations of the tested compound (10, 20, 50, 100 and 200 μg/ml), the cells were incubated for an additional 48 h at 37°C. After incubation, the medium was removed, and cells in each well were incubated with 50 μl of MTT solution (5 mg/ml) for 4 h at 37°C. MTT solution was then discarded, and 50 μl of DMSO were added to dissolve insoluble formazan crystal. Optical density was measured at 540 nm. Data were obtained from triplicate wells. The proliferation inhibitory effect was determined with regard to the negative control (vehicle treated cells).

### DNA fragmentation analysis

DNA fragmentation was analysed by agarose gel electrophoresis as described by Wang *et al.* (2002). TK6 cells (1.5 10^6^ cells/ml) were exposed to tested compound at a concentrations of 2.5, 5 and 10 μg/ml for 24 and 48 h and harvested by centrifugation. Control cells were also treated with 0.5% of DMSO.

Cell pellets were resuspended in 200 μl of lysis buffer (50 mM Tris–HCl, pH 8.0, 10 mM EDTA, 0.5% N-Lauroyl Sarcosine Sodium Salt) at room temperature for 1 h, then centrifuged at 12 000 g for 20 min at 4 C. The supernatant was incubated overnight at 56°C with 250 μg/ml proteinase K. Cell lysates were then treated with 2 mg/ml RNase A and incubated at 56 C for 2 h. DNA was extracted with chloroform/phenol/isoamyl alcohol (24/25/1, v/v/v) and precipitated from the aqueous phase by centrifugation at 14 000 g for 30 min at 0°C. The solution recuperated was transferred to a 1.5% agarose gel and electrophoresis was carried out at 67 V for 3/4 h with TAE buffer (Tris 2 M, sodium acetate 1 M, EDTA 50 mM) as the running buffer. DNA in the gel was visualized with ethidium bromide (0.5 μg/ml) under UV light [22].

### Western blot analysis

Cells, treated with different concentrations of DGA (2.5, 5 and 10 μg/ml) for 6, 24 and 48 h as well as control cells treated with 0.5% DMSO, were lysed with a lysis buffer (62.5 mM Tris HCl and 6 M urea, pH = 6.8). Protein concentrations were determined in cell lysates using the Bradford method [23]. Equal amounts of proteins were separated on sodium dodecyl sulfate polyacrylamide gel electrophoresis (SDS-PAGE), and transferred onto PVDF membrane, which was then blocked with 5% of non-fat milk in 0.1% Tween 20-Phosphate buffer salin (PBST) overnight at 4°C. Membranes were then incubated with a primary antibody anti-PARP at a 1:100 dilution for 2 h at room temperature. The membrane was then washed and incubated with a goat anti-mouse alkaline phosphatase-conjugated antibody at 1:7500 dilution for 1 h. Next, the membrane was washed and the chromogenic substrate BCIP/NBT was added to localise antibody binding proteins.

### The study of caspase-3 and caspase-8 activities

Cells were cultured (10^6^ cells/ml) in 25 cm^2^ flasks for 24 h in the absence or the presence of 2.5, 5 and 10 μg/ml of DGA at 37°C. Controls were performed at the same time with 0.5% DMSO. Cells were harvested and centrifuged at 600 × *g* and the pellets were washed with PBS, then incubated in ice cold lysis buffer for 15 min, then centrifuged at 16000 × *g* for 20 min. Supernatants (cell extracts eventually containing caspase-3 and caspase-8) were retrieved and aliquots corresponding to 50 μg total protein were incubated along with acetylated tetrapeptide (Ac-DEVD) substrate labelled with the chromophore *p*-nitroaniline (pNA) in the presence of caspase buffer in a 96-well flat bottomed microplate. In the presence of active caspase-3 and caspase-8, cleavage and release of pNA from the substrate occurs. Free pNA produces a yellow color, detected spectrophotometrically at 405 nm. Lecture of absorbance at 405 nm was done against a blank performed at the same time and containing assay buffer and substrate but without cell lysate. A standard curve was realized in order to determine the correspondence between absorbance and pNA concentration, then the results were expressed as caspase-3 and caspase-8 specific activity (μmol pNA per min/ml protein) calculated as indicated by the manufacturers.

### Statistical analysis

All tests were carried out in triplicate and the results were presented as means ± SD. The data were tested for statistical differences by one-way ANOVA followed by Dunett test using STATISTICA (Version 6.0, Statsoft, Inc.) to compare data of control (untreated cells) to those of cells treated by different extracts. Statistical differences were determined at the *P* < 0.05.

## Results

### Evaluation of antiproliferative activity

**Figure 2:**
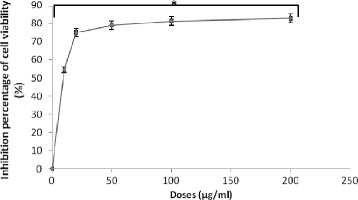
**Inhibitory effect of digallic acid on the viability of TK6 cells.** *Significant from control (*p* < 0.05).

Human lymphoblastoid TK6 cells were treated with the compound isolated from *P. lentiscus* fruits, for 48 h at 37°C. Antiproliferative activity of the compound was evaluated by the MTT assay. The results, summarized in Figure 2, showed that the DAG inhibited cell proliferation. The IC_50_ value was 8.5 μg/ml.

### Induction of apoptotic DNA fragmentation by DGA

**Figure 3:**
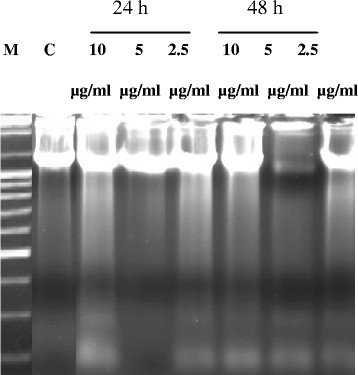
**DNA electrophoretic profile on 1.5% agarose gel of TK6 cells treated with different concentrations of DGA for 24 and 48 h.** C: negative control; untreated cells M: molecular weight markers (bp).

The fragmentation of TK6 cell DNA was detected on a 1.5% agarose gel electrophoresis after exposing 1.5 x 10^6^ cells to 0, 2.5, 5 and 10 μg/ml of DGA during 24 h and 48 h. Examination of cell DNA electrophoretic profiles revealed a ladder formation, which is characteristic of apoptosis (Figure 3). At exposure to 2.5-10 μg/ml with DGA during 24 h and at exposure to 2.5-10 μg/ml during 48 h, a ladder DNA profile was clearly observed in TK6 cells. Whereas control cells did not provide any ladder DNA profile. We deduce that DGA compound from *Pistascia Lentiscus fruits* induces apoptosis in TK6 cells.

### Effect of digallic acid on the proteolysis of PARP

DNA fragmentation is often associated with the activation of a family of cysteine proteases, the caspases. Caspase 3, in particular, seems to play an important role in several models of apoptosis [10,23]. To confirm the apoptotic process of the observed DNA fragmentation, we also investigated, the enzymatic activation of apoptotic proteins by measuring the cleavage of PARP, which is a caspase-3 substrate.

**Figure 4:**
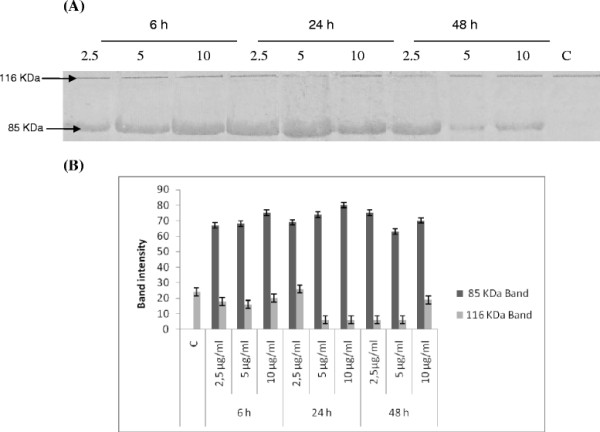
**(A) Changes in expression of apoptotis-related protein in response to treatment with digallic acid.** TK6 cells were treated with 2.5, 5 and 10 μg/ml of digallic acid for 6, 24 and 48 h. Protein extracts were subjected to western blotting to determin immunoreactivity levels of PARP, as described in methods section. PARP 116 KDa and 85 KDa bands are shown. **C**: Cells treated with 0.5% DMSO. (**B**) Quantification by scanning densitometry of PARP bands intensity*.

As shown in Figure 4, when cells were treated with *P.lentiscus* compound, an increase in the formation of 85 kDa fragment and a decrease or a total disappearence of the 116 kDa band were observed. The addition of DGA induces cleavage of 116 kDa PARP into fragments of 85 and 31 kDa in an inversely concentration- dependant manner, at the three tested time incubation.

### Caspase-3 and caspase-8 activation assay

**Figure 5:**
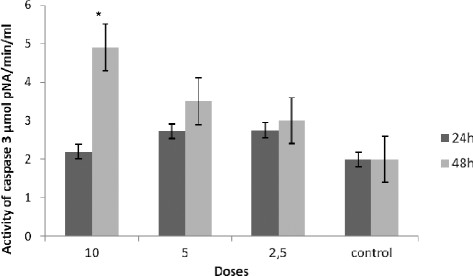
**Effect of digallic acid (DGA) on caspase-3 activity in TK6 cells.** Lysates prepared from cells treated with the digallic acid for 24 h and 48 h were assayed for *in vitro* caspase-3 activity. The rate of cleavage of the caspase substrate DEVD-pNA was measured at 405 nm. The results are presented as the mean ± SD. The experiments were done in triplicate. (*) p < 0.05 means a significant difference between the untreated and treated cells. Control: cells were treated by the vehicle only*.

**Figure 6:**
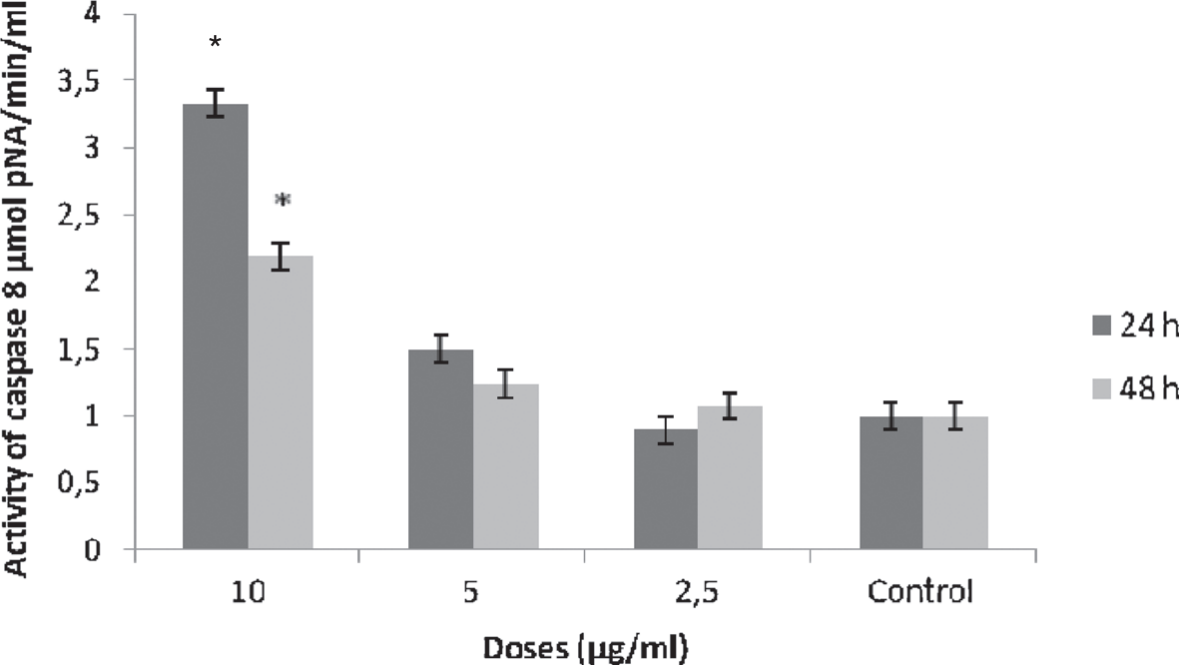
**Effect of digallic acid (DGA) on caspase-8 activity in TK6 cells.** Lysates prepared from cells treated with the digallic acid for 24 h and 48 h were assayed for *in vitro* caspase-8 activity. The rate of cleavage of the caspase substrate DEVD-pNA was measured at 405 nm. The results are presented as the mean ± SD. The experiments were done in triplicate. (*) p < 0.05 means a significant difference between the untreated and treated cells. Control: cells were treated by the vehicle only.

The cellular pathway of DGA induced cell death was examined by assessing caspase-3 and caspase-8 activities. Following, 24 h and 48 h treatment of TK6 cells with various concentrations of DGA, caspase-3 and caspase-8 activities were measured and compared with control cells. As shown in Figure 5 and Figure 6, TK6 cells treated with DGA for 24 and 48 h, showed a significant concentration- depending increase of caspase-3 and caspase-8 activities.

At the highest concentration 10 μg/ml, the values of caspases 3 activity were 2.2 and 4.9 μmol pNA/min/ml at respectively 24 h and 48 h incubation. At this concentration the compound showed a significant induction of caspase 3 activity compared to untreated cells (2 μmol pNA/min/ml). In the same way, DGA showed the highest caspase 8 induction activity at the same concentration (10 μg/ml) after 24 h of treatment with a value of 3.33 pNA/min/ml. These results suggest that apoptosis induced by the tested polyphenol may occur through the activation of common executors of apoptosis, such as caspase-3 through the activation of caspase-8.

## Discussion

In recent years, the role of lifestyle and dietary behavior in reducing cancer risk has drawn widespread attention based on the geographic differences in cancer incidence and mortality. Natural compounds have been adopted increasingly in the field of chemoprevention, as well as the synthetic chemicals, that have been identified for inhibiting or reversing carcinogenesis [24,25]. Cancer preventive phytochemicals have been shown to suppress or block cancer progression by a variety of mechanisms including; acting as anti-proliferative agents or as antioxidants [26]. On the basis of this regard, extensive investigations on phenolic acids have shown promising results against different cancers including human leukemia, gastric cancer, colon cancer, *etc.*[27,28].

Digallic acid, is a polyhydroxyphenolic compound, widely distributed in various plants and fruits, such as *Pistacia lentiscus* and *Myrtus communis* from Tunisian flore [19,29]*,* green tea, apple peels, grapes, strawberries, lemons and in red and white wine. Our previous studies established that digallic acid showed significant antioxidant and antimutagenic activities [16].

In this study, we found that DGA inhibited cell growth of human lymphoblastoid cell lines with an IC_50_ value of 8.5 μg/ml and that cell death occurs by apoptosis as shown by oligonucleosomal DNA cleavage (“DNA ladder”) (Figure 3).

Furthermore, Isuzugawa *et al.* (2001) and Inoue *et al.*(1995) have demonstrated that GA selectively induces cancer cell death by apoptosis; however, gallic acid shows no cytotoxicity against normal cells [30,31].

Apoptosis produced the typical pattern of apoptotic PARP cleavage: a catalytically active band of intact PARP at 116 kDa, and an active band at 85 kDa corresponding to the apoptotic cleavage product of PARP. PARP is proteolytically cleaved during apoptosis by caspase-3 [32] which reduces PARP’s enzymatic activity [33], thereby inhibiting DNA repair. As shown In Figure 4 DGA- treated TK6 cells show a dose-dependent increase in the cleavage of PARP (85 kDa) which might indicate a breakdown in the DNA repair function.

Indeed, Caspases are synthesized as inactive pro-enzymes, and their activation during apoptosis results in cleavage at specific aspartate cleavage sites [34]. The downstream signals during apoptosis are transmitted *via* caspases. Upon conversion from pro- to active forms, caspases mediate the cleavage of PARP, followed by DNA fragmentation. The DNase responsible for the fragmentation is reportedly activated directly by caspase-3 [33,34]. Caspase-3 is one of the key executioners of apoptosis, being responsible either partially or totally for the proteolytic cleavage of many key proteins, such as the nuclear enzyme PARP. Thus, PARP is known to be cleaved in the execution phase of apoptosis.

In the present study, we showed that DGA induced the activation of caspase-3 in a dose-dependent manner (Figure 5) leading to the cleavage of PARP. This finding indicates that activation of the caspase-3 pathway may mediate the proapoptotic activity of the tested compound.

Caspases-8 is synthesized as inactive proenzymes and becomes activated either by oligomerization in a large multimeric complex, or alternatively *via* proteolytic cleavage, which applies for effector caspases such as caspase-3 [35]. Once activated, they cleave various substrates in the cytoplasm or nucleus causing characteristic morphological features of apoptotic cell death [34]. In the extrinsic apoptosis pathway, stimulation of death receptors of the tumor necrosis factor (TNF) receptor superfamily, *e.g.* CD95 (APO-1/Fas) or TRAIL receptors, results in activation of the initiator caspase-8, which in turn can directly cleave downstream effector caspases such as caspase-3 [35].

Our result demonstrates that caspase-8 was activated by DGA in a dose and time depending manner.

According to Chen *et al.* (1999) [36] and Liang *et al.* (1999) [37] a pure compound which has two gallic acid moieties, exhibited the strongest anti-proliferative activity on tumor cells by report to a compound which has no gallic acid moiety [36,37]. This is in accordance with our result as we obtained a high cytotoxicity against TK6 cells, in the presence of DGA.

Our results revealed the importance of galloyl moieties in inducing apoptosis.

This finding is in accordance with those reported by Pan *et al.* (1999) who demonstrates that a compound isolated from hydrolyzed tannin, which has five gallic acids, was the most potent apoptosis inducer among tested polyphenols.

According to his study he concludes that gallic acid moieties are important for the exhibited apoptosis-inducing potency of these polyphenols [38].

Besides, Haslam *et al.* (1996) showed that the molecular size of the polyphenol is important; in the galloyl series, the efficacy of binding increases as the number of galloyl groups increases in the order of: tri tetra penta [39]. Moreover, Sakagami *et al.* (1995) reported a high induction of DNA fragmentation in the presence of gallic acid a component unit of tannin [40].

## Abbreviations

DGA: Digallic acid; DMSO: Dimethylsulfoxide; MTT: 3-(4,5-dimethylthiazol-2-yl)-2,5-diphenyltetrazolium bromide; PARP: Poly (ADP-ribose) polymerase.

## Competing interests

The authors declare that they have no competing interests.

## Authors’ contributions

BW: Was responsible for the conception and design, testing and data acquisition, analysis and data interpretation and drafted the manuscript. BJ: made contribution to the study of caspase activities. SI: made contribution to cell culture and the study of the cytotoxicity and the DNA fragmentation. GK: made substantial contribution to conception and revised it critically for Important intellectual content. CGL:made substantial contribution to conception and revised it critically for important intellectual content. All authors read and approved the final manuscript.
